# Hypoxia Induces Apoptosis through HIF-1*α* Signaling Pathway in Human Uterosacral Ligaments of Pelvic Organ Prolapse

**DOI:** 10.1155/2017/8316094

**Published:** 2017-11-02

**Authors:** Xinrui Zhao, Congcong Ma, Rui Li, Jing Xue, Lidong Liu, Peishu Liu

**Affiliations:** Department of Obstetrics and Gynecology, Qilu Hospital of Shandong University, 107 Wenhua Xi Road, Jinan, Shandong 250012, China

## Abstract

The purpose of this study is to evaluate the expression of hypoxia-inducible factor-1*α* (HIF-1*α*) in women uterosacral ligament tissues with pelvic organ prolapse and women with normal uterine support structures and illuminate its relationship with apoptosis. Samples were collected from 38 women with pelvic organ prolapse and 31 age matched controls. The expression levels of HIF-1*α* and BNIP3 in the uterosacral ligaments were measured using immunohistochemistry, qRT-PCR, and Western blot. To assess apoptosis we performed TUNEL assay and Western blot analyses. Lastly, the short form of the Pelvic Floor Impact Questionnaire-7 (PFIQ-7) was used to evaluate prognosis of surgical patients and twenty patients finished the follow-up. The expressions of HIF-1*α* and BNIP3 in the uterosacral ligaments were significantly higher in patients with pelvic organ prolapse than in control group. Pearson's correlation test revealed significant positive correlations between HIF-1*α* and apoptosis index. Similarly, Western blot analysis showed the expression of proapoptosis proteins (Bax and Bad), Cytochrome-c, cleaved caspase-3, and caspase-9 in patients with pelvic organ prolapse was upregulated. The PFIQ-7 scores were higher in HIF-1*α* positive group than in the negative group. Hypoxia may contribute to the pathological process of pelvic organ prolapse by increasing apoptosis via activating HIF-1*α* signaling pathway.

## 1. Introduction

Pelvic organ prolapse (POP), the fall of the female pelvic organs (vagina, uterus, bladder, and/or rectum) into or through the vagina, is a common and costly condition in elderly women [1]. The estimated lifetime risk of surgery for POP is 12.6% by the age of 80 years [2]. Over 200,000 surgeries are performed annually in the United States for POP, costing more than 1 billion dollars a year [3, 4]. Although several risk factors for POP such as advancing age, vaginal childbirth, increasing body-mass index, family history of POP, and chronic intra-abdominal pressure are well established [5–7], the underlying molecular mechanism remains unknown.

Pelvic organ support is mainly provided by the connective tissues (endopelvic fascia and ligaments) and the levator ani muscles [5]. The uterosacral ligaments (USLs) are important parts of the support system [8]. Traditionally, research on the pathophysiology of POP has centered on alteration of extracellular matrix (ECM), such as decreased amount of collagen, altered collagen metabolism, and increased breakdown of collagen and elastin [9–12]. Recently, studies have suggested that apoptosis may be a contributor in the development of pelvic floor disorders [13, 14]. Apoptosis, programmed cell death, is fundamental to many physiologic processes, such as embryogenesis and tissue remodeling during healing [15]. Pelvic trauma related childbirth; pelvic surgeries; or even chronic mechanical stress may increase apoptosis and lead to abnormal tissue remodeling. Some studies have proposed the hypothesis that hypoxia may cause the apoptosis of the USLs [16].

Hypoxia triggers the expression of hypoxia-inducible factor-1*α* (HIF-1*α*) protein and increases its nuclear translocation [17]. HIF-1*α* may play a role in cell death or cell survival via upregulating downstream proteins such as Bcl-2/adenovirus E1B 19-kDa protein (BNIP3). BNIP3, a member of BH3-only subfamily of Bcl-2 superfamily, belongs to proapoptotic proteins. It localizes on the outside of the mitochondrial membrane. Once being stimulated, BNIP3 leads to the activation of Bax and/or Bak, opening of the mitochondrial permeability transition pore and finally apoptosis [18–20]. Thus, we hypothesized that hypoxia may contribute to the pathological process of POP via HIF-1*α* pathway.

Our study sought to explore the expression of HIF-1*α* and its target gene, BNIP3, in the USLs. In addition, this study also investigated the rate of cell apoptosis and examined expression of the apoptosis-related proteins Cytochrome-c (Cyto-c), caspase-3, caspase-9, and Bcl-2 family in order to determine whether hypoxia influences cell apoptosis in USLs.

## 2. Materials and Methods

### 2.1. Study Population and Sample Collection

This study was approved by the ethics committee of the Qilu Hospital of Shandong University. Written informed consent was collected from each eligible patient. All participating women were followed up by reviewing their medical records and telephoning.

A total of 69 women were enrolled from the Department of Obstetrics and Gynecology, Qilu Hospital of Shandong University, between April 2010 and December 2016. The patient group consisted of 38 women, who underwent hysterectomy as part of pelvic reconstruction surgery for POP that was stage II, III, or IV according to the POP quantitative (POP-Q) examination [21]. In addition, the control group consisted of 31 age matched women with stage 0 or I POP-Q who underwent operations for benign gynecologic indications, including cervical intraepithelial neoplasia and dysfunctional uterine bleeding. Women who had pelvic infections, cancer, hormone replacement therapy (HRT), cardiovascular diseases, diabetes mellitus, and other endocrine dysfunction, neurodegenerative disorders, and immunological or inflammatory disorders were not included in either group. Furthermore, menopause was defined as the cessation of menses for at least 1 year.

At surgery, tissue samples measuring 0.5 × 1.0 × 1.0 cm were obtained from the right side USLs closed to the cervix, where the ligament is consistently identifiable. Half of the specimens were immediately snapped frozen in liquid nitrogen and kept at −80°C until RNA extraction and Western blot analysis were processed. The other half of the specimens were fixed in formalin and embedded in paraffin for immunohistochemistry and TUNEL assay.

### 2.2. Immunohistochemical Staining and Evaluation

Immunohistochemical analysis of all subjects was performed on 4-*μ*m thick tissue sections using the standard PV two-step staining kit (ZSGB-BIO, Beijing, China). According to the manufacturer's instruction, the primary mouse anti-HIF-1*α* antibody (1 : 200; ab150595, Abcam, Cambridge, UK) and rabbit anti-BNIP3 (1 : 100; ab150595, Abcam, Cambridge, UK) antibody incubated tissue sections overnight at 4°C. The primary antibody was replaced with Tris-buffered saline to act as the negative control. Subsequently, these sections were exposed by DAB and hematoxylin. Staining intensity was scored as follows: absent (0), weak (1), moderate (2), or strong (3). Quantifications were recorded as follows: <25% positive cells (0), 25–50% (1), 51–75% (2), and >75% (3). The final score was the multiplication of the staining intensity and quantification. A final score of 0-1 was classified as negative, and >1 was considered positive.

### 2.3. TUNEL Assay

The TUNEL (TdT-mediated digoxigenin-dUTP nick-end labeling) method was carried out with a commercially available in situ apoptosis detection kit (Solarbio, Beijing, China). Staining was performed according to the manufacturer's protocol. DAPI was used to visualize all nuclei. Cells with TUNEL-positive nuclei (red) were detected by fluorescence microscopy (Olympus). The percentage of apoptotic cells was calculated by dividing the number of TUNEL-positive cells by the total number of cells visualized in the same field.

### 2.4. Quantitative Real-Time Reverse Transcription Polymerase Chain Reaction (Real-Time RT-PCR)

The total RNA of 12 ligament specimens obtained from patients with POP and 12 from patients with non-POP controls was extracted using TRIzol reagent (Life Technologies, Carlsbad, CA, USA). Then, the RNA was transcripted into cDNA using M-MLV reverse transcriptase (Invitrogen, Shanghai, China) according to the manufacturer's instructions. PCR was performed on StepOne (Applied Biosystems, Shanghai, China) and data quantification using the 2^−ΔΔCt^ method. The following primers (BioSune Biotechnology Co., Ltd., Shanghai, China) were used: GAPDH (used for normalization), forward 5′-GCA CCG TCA AGG CTG AGA AC-3′ and reverse 5′-TGG TGA GGT AGA CGC CAG TGG A-3′; HIF-1*α*, forward 5′-ATC CAT GTG ACC ATG AGG AAA TG-3′ and reverse 5′-TCG GCT AGT TAG GGT ACA CTT C-3′; BNIP3, forward 5′-CAG GGC TCC TGG GTA GAA CT-3′ and reverse 5′-CTA CTC CGT CCA GAC TCA TGC-3′.

### 2.5. Western Blot Analysis

Twenty-four samples selected from the patient and control groups were homogenized in RIPA buffer (Beyotime Institute of Biotechnology, Haimen, China) with 1% phenylmethylsulfonyl fluoride (PMSF) and 1% NaF for 1 h and centrifuged at 12000 gravities for 15 minutes at 4°C. Equal amounts of protein (30 *μ*g) were loaded and separated by 10% sodium dodecyl sulfate-polyacrylamide gel electrophoresis and transferred to polyvinylidene fluoride membranes (Millipore). The membranes were blocked with 5% nonfat dry milk for 1 hour and incubated with anti-HIF-1*α* and anti-BNIP3 (Abcam); anti-Bcl-2, anti-Bcl-xl, anti-Bax, and anti-Bad (Santa Cruz Biotechnology); anti-Cytochrome-c, anti-caspase-3, anti-cleaved caspase-3, anti-caspase-9, anti-cleaved caspase-9, and anti-GAPDH [Cell Signaling Technology (CST)] antibodies overnight at 4°C. The membranes were then incubated with respective secondary antibodies (Millipore) and visualized by enhanced chemiluminescence (ECL) using ImageQuant LAS 4000 (GE Healthcare Life Sciences, Logan, UT, USA). The GAPDH band served as control. The results were analyzed by ImageJ software.

### 2.6. Postoperative Follow-Up

We selected patients to follow-up, who underwent transvaginal total hysterectomy plus colporrhaphy anterior-posterior surgery about 3 years ago. Twenty women were divided into two groups according to the immunoreactivity of HIF-1*α* (including 8 cases of HIF-1*α* negative, 12 cases of HIF-1*α* positive). All women filled in the short forms of the Pelvic Floor Impact Questionnaire-7 (PFIQ-7) [22].

### 2.7. Statistical Analysis

GraphPad Prism 5.01 (GraphPad Software, USA) was used for statistical analysis. In the present study, data were expressed as mean ± standard deviation (SD), and statistical comparisons were performed using Student's *t*-test or the Chi-squared test. Correlation analyses were performed with Pearson's correlation test. *P* values < 0.05 were considered statistically significant.

## 3. Results

### 3.1. Clinical Characteristics of Patients with POP and Control Groups

As listed in Table 1, the clinical characteristics of patients with POP and control groups were matched in terms of demographic and clinical characteristics. No significant differences were observed in age, body-mass index, parity, or menopausal status between the two groups.

### 3.2. The Expression of HIF-1*α* and BNIP3 in USL Tissues of POP and Control Groups

**Figure 1:**
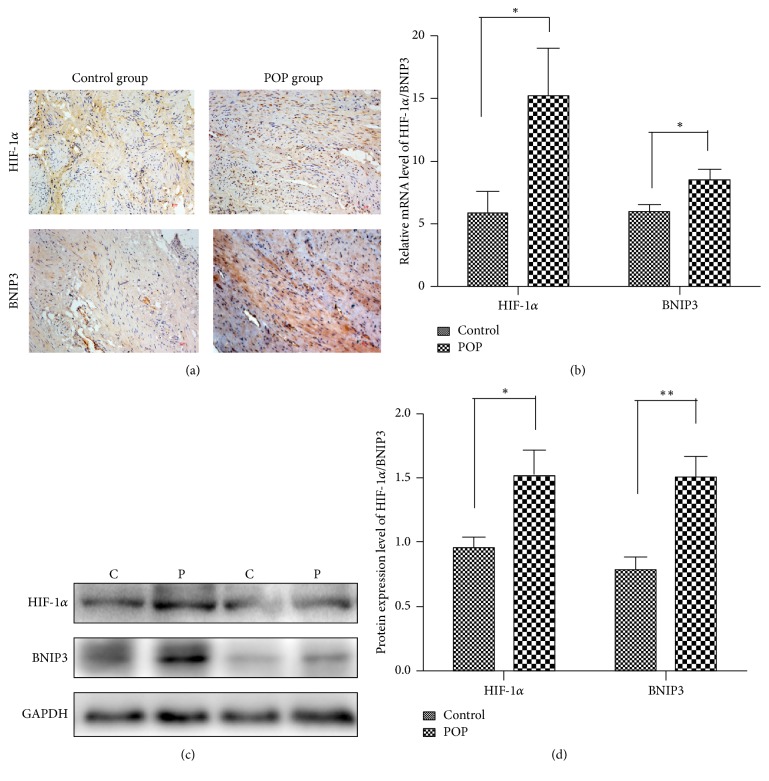
The expression of HIF-1*α* and BNIP3 are upregulated in USL tissues of the POP group. (a) Representative immunohistochemical staining of HIF-1*α* and BNIP3 in the uterosacral ligaments in control and POP group (magnification, ×200; scale bar = 10 *μ*m). (b) Quantification of the expression of HIF-1*α* and BNIP3 between the POP group and the control group at the mRNA level. ^*∗*^*P* < 0.05. (c and d) HIF-1*α* and BNIP3 were detected by Western blot analysis at the protein level. ^*∗*^*P* < 0.05. ^*∗∗*^*P* < 0.01.

Using immunohistochemical assay, we found that the immunoreactivity of HIF-1*α* in USL tissues of the POP group was significantly higher than the control group (1.477 ± 0.779, versus 2.668 ± 1.542, resp.; *P* < 0.01). BNIP3 showed the same results (1.523 ± 0.615, versus 3.084 ± 1.618, resp.; *P* < 0.001; Figure 1(a)). Correlation analyses revealed a significant positive correlation between HIF-1*α* and BNIP3 (Pearson correlation coefficient 0.77, *P* < 0.001). Similarly, qRT-PCR and Western blot analysis revealed the expression of HIF-1*α* and BNIP3 was increased in the level of mRNA and protein, respectively (Figures 1(b)–1(d)).

### 3.3. Relationship between the Expression of HIF-1*α* in POP USL Tissues and Clinical Characteristics

Next, we performed the immunohistochemical staining results of HIF-1*α* in Table 2. We found that the immunoreactivity of HIF-1*α* in stage III group was significantly higher than stage II group (*P* = 0.001), and no significant differences were found between stage III and stage IV group (*P* = 0.053). In addition, the expression level of HIF-1*α* in USL tissues was not correlated with age (*P* = 0.259) and menopausal status (*P* = 0.311).

### 3.4. The Level of Apoptosis in USLs of POP Patients and Controls

**Figure 2:**
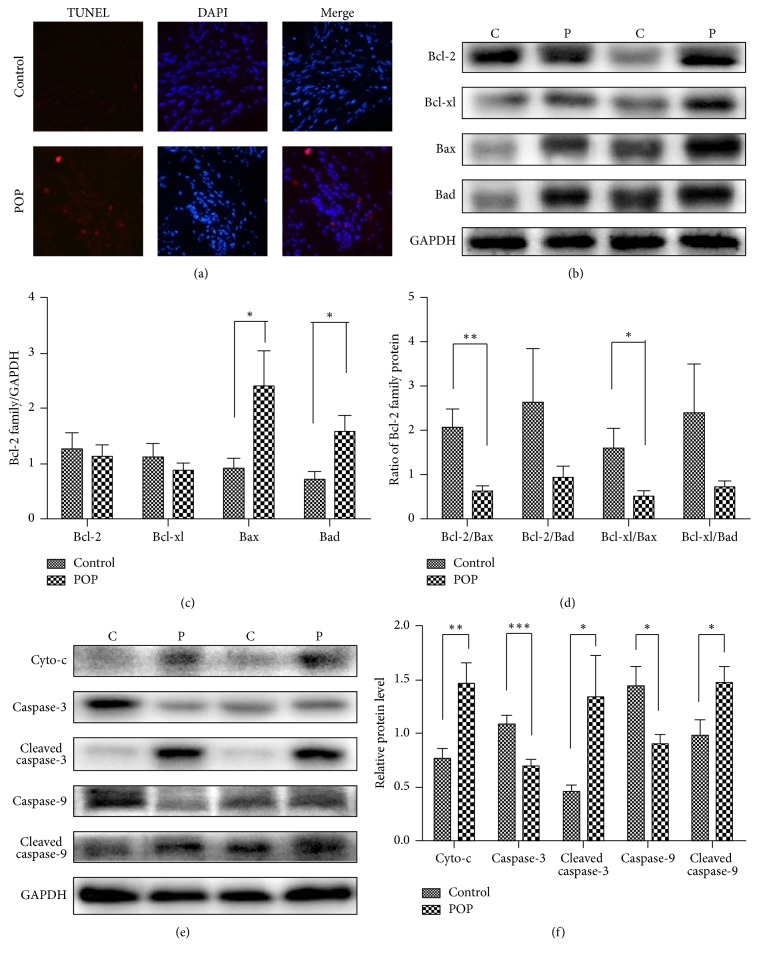
TUNEL assay and expression of apoptotic proteins in USL tissues of POP patients and control group. (a) TUNEL assay. (b) The protein expression of the Bcl-2 family in the POP group and control group. (c) Quantification of the expression of Bcl-2 family in USL tissues showed in (b) ^*∗*^*P* < 0.05. (d) Quantification of the antiapoptotic/proapoptotic proteins expression ratio showed in (b) ^*∗*^*P* < 0.05 and ^*∗∗*^*P* < 0.01. (e) The protein expression of the caspase family in the POP group and control group. (f) Quantification of the expression of Cyto-c and caspase family showed in (e) ^*∗*^*P* < 0.05; ^*∗∗*^*P* < 0.01; ^*∗∗∗*^*P* < 0.001. The results are presented as the mean ± SD.

The TUNEL assay demonstrated apoptosis in the USL cells in POP and control groups (Figure 2(a)). The percentage of apoptosis cells was significantly higher in POP group than in controls (16.42% ± 7.94% versus 7.91% ± 5.56%, *P* < 0.01), and significant positive correlation was detected between the immunoreactivity of HIF-1*α* and the percentage of apoptosis cells (*r* = 0.60, *P* < 0.01).

The expression of Bax and Bad in USL tissues from the POP group was higher than that in the control group (*P* < 0.05). However, the expression of Bcl-2 and Bcl-xl in the POP group was not significantly lower than the control ones (Figures 2(b) and 2(c)). We also examined the ratio of antiapoptotic proteins to proapoptotic proteins. The Bcl-2/Bax, Bcl-2/Bad, Bcl-xl/Bax, and Bcl-xl/Bad ratios in USL tissues of the POP group were lower than the control group, but only the Bcl-2/ Bax ratio (*P* < 0.01) and Bcl-xl/Bax ratio (*P* < 0.05) were significantly different between two groups (Figure 2(d)). Similarly, Western blot analysis showed the expression of procaspase-3 and procaspase-9 decreased, and the expression of Cyto-c, cleaved caspase-3, and caspase-9 increased in patients with POP (Figures 2(e) and 2(f)).

### 3.5. PFIQ-7 Scores of Postoperative Patients

The PFIQ-7 presents seven questions with urinary impact, colorectal-anal impact, and pelvic organ prolapse impact. Each aspect has four answers (not at all = 0; somewhat = 1; moderate = 2; a lot = 3). The final score was calculated when average of every column was multiplied by 33.3, and total score ranged from 0 to 300 [23]. The total scores were significantly different between groups, with higher scores in HIF-1*α* positive group (38.49 ± 19.63 versus 17.26 ± 10.78, *P* < 0.05).

## 4. Discussion

Female pelvic floor tissues are subject to multiple mechanical stresses, including pregnancy, childbirth, defecation, cough, and normal gravity, which contribute to the increase in abdominal pressure [24]. This pressure extension of pelvic floor expansion is thought to result in hypoxia and production of free radicals and reactive oxygen species [25].

We demonstrated that the expression of HIF-1*α* and its target gene, BNIP3, was significantly upregulated in USL tissues in the POP group. Furthermore, there was a positive correlation between the expression of HIF-1*α* and apoptosis index in USLs. We also preliminarily demonstrated the HIF-1*α* expression is associated with life quality after operation. To the best of our knowledge, the present study is the first to report the effects of hypoxia on apoptosis in USL tissues with POP.

When cells are exposed to chronic or extreme hypoxia, the expression of HIF-1*α* was increased and resulted in apoptosis. As a transcription factor, HIF-1*α* is involved in cell death by activating prodeath genes, such as BNIP3 [26]. BNIP3 is a BH3-only Bcl-2 family member regulated by HIF-1*α*, and it has been identified as one of the most prominent hypoxia responsive genes [27]. The expression of BNIP3 can be upregulated under hypoxia in cell lines such as carcinomas [28, 29], fibroblasts [30], and macrophages [31]. We have also found that expression of HIF-1*α* and BNIP3 in USLs with POP was significantly increased both in mRNA and in protein levels. Although the results of immunohistochemistry revealed that the expression of HIF-1*α* was not associated with age and menopausal status, expression level was higher in the severe prolapse group compared to the mild prolapse group. We could see a significant difference in the HIF-1*α* levels between stage II and stage III group, but not between stage III and stage IV group. In general, the patients of stage IV had stopped developing when they underwent the operations. The patients of stage II and stage III, however, are in a period of aggravation. It also proves that HIF-1*α* has participated in the development of POP.

Takacs et al. compared 12 women who underwent surgery for either POP repair or benign gynecological disease and found a significant increased apoptosis in smooth muscle cells [32]. Comparison between the smooth muscle and apoptotic cells in the USL tissues of women with and without POP demonstrated an increased apoptosis in women with POP [33]. Consistently, our results exhibited an increase in apoptotic index by TUNEL analysis in the USLs of patients with POP. In addition, correlation analyses revealed a significant correlation between the expression of HIF-1*α* and apoptosis index, indicating that the increased apoptosis noted in the pelvic connective tissues of POP patients was closely related to hypoxia.

The BH3 domain of BNIP3 plays an important role in cell death, as well as in mediating heterodimerization with antiapoptotic proteins or proapoptotic proteins, which regulate cell death [34]. We found that the expression of the proapoptotic proteins was Bax and Bad was increased and the ratios of Bcl-2/Bax and Bcl-xl/Bax were reduced in USL tissues in the POP group compared with the non-POP group. Lower ratios indicate higher sensitivity to apoptosis. These findings were consistent with those of Wen et al., who found higher expression of proapoptotic proteins in vaginal tissues from women with POP [14]. However, Saatli et al. found that the expression of both the antiapoptotic genes and the proapoptotic genes was increased in vaginal and USL tissues in the POP group compared with the control group [35]. Diversity of age, menopausal status, and histologic type in the different patient groups may provide the difference.

In recent years, the mechanisms underlying the intrinsic [36] and extrinsic [37] apoptotic pathways have been clarified [38]. The binding of death-inducing ligands to cell-surface death receptors can stimulate the extrinsic pathway. DNA damage, growth factor deprivation, and oxidative stress can activate the intrinsic pathway. Initiation of the intrinsic pathway leads to mitochondrial depolarization, which allows the release of Cytochrome-c. In turn, Cytochrome-c engages apoptotic protease activating factor 1 (APAF 1) and forms the apoptosome in the presence of deoxyadenosine triphosphate. The apoptosome activates caspase-9, which activates caspase-3 and induces apoptosis [39]. We investigated the expression of the Bcl-2 family, key regulators of mitochondrial apoptosis, and demonstrated the expression of proapoptotic proteins was increased and the ratio of antiapoptotic to proapoptotic proteins was downregulated in the USL tissues of patients with POP compared to females with non-POP. Consistent with that, our results exhibited the expression of Cyto-c, cleaved caspase-3, and caspase-9 in the USLs of patients with POP was increased. Therefore, we believed that hypoxia induced intrinsic apoptotic pathway, which led to attenuation of pelvic support tissues as a reasonable mechanism in pathogenesis and prognosis of POP.

Limitations to our study include small sample size of USL tissues and follow-up patients. During the follow-up period, many patients were lost for various reasons, such as death. Therefore, the sample size of follow-up was further reduced. At present, there is no established animal model or fibroblasts cell line of uterine ligaments to be experimented on, which limited the development of POP research. We will train primary fibroblasts from the USL ligament tissues to further clarify the pathogenesis of POP.

In conclusion, hypoxia resulted in cell death with apoptosis in human USL tissues via activating HIF-1*α* signaling pathway. The pathogenesis and prognosis of POP may depend on HIF-1*α* signaling pathway, which further modulated the expression of the Bcl-2 family proteins and the caspase family proteins, such as BNIP3, Bax, Bad, Cyto-c, caspase-3, and caspase-9. Therefore, approaches to modulate this undesirable condition seem to be useful in preventing or delaying the progression of POP.

## Figures and Tables

**Table 1:** Clinical characteristic of patients with POP and control groups.

	POP (*n* = 38)	Control (*n* = 31)	*P* value
Age (years, mean ± SD)	56.61 ± 7.41	53.65 ± 6.54	0.09
BMI (mean ± SD)	24.26 ± 3.11	23.48 ± 3.44	0.33
Parity (mean ± SD)	2.24 ± 0.97	1.87 ± 0.88	0.11
Menopause (%)	63.16	45.16	0.13
POP-Q stage			
0-I	0	31	
II	10	0	
III	20	0	
IV	8	0	

**Table 2:** Relationship between HIF-1*α* expression in POP USL tissues and clinical characteristics.

Clinical factors	Number	Expression of HIF-1*α*		*P* value
		Negative	Positive	
Age (years)				
≤50	9	5	4	
>50	29	10	19	0.259
Menopausal status				
Before menopause	14	7	7	
Menopause	24	8	16	0.311
POP-Q stage				
II	10	8	2	
III	20	3	17	0.001
IV	8	4	4	0.053
